# The Role of BPIFA1 in Upper Airway Microbial Infections and Correlated Diseases

**DOI:** 10.1155/2018/2021890

**Published:** 2018-09-03

**Authors:** Yung-An Tsou, Min-Che Tung, Katherine A. Alexander, Wen-Dien Chang, Ming-Hsui Tsai, Hsiao-Ling Chen, Chuan-Mu Chen

**Affiliations:** ^1^Department of Otolaryngology Head and Neck Surgery, School of Medicine, China Medical University Hospital, Taichung 404, Taiwan; ^2^Department of Life Sciences, National Chung Hsing University, Taichung 402, Taiwan; ^3^Department of Surgery, Tungs' Taichung MetroHarbor Hospital, Taichung 435, Taiwan; ^4^Graduate Institute of Clinical Medicine, Taipei Medical University, Taipei 110, Taiwan; ^5^Department of Biology, Washington University in St. Louis, St. Louis, Missouri 63130, USA; ^6^Department of Sport Performance, National Taiwan University of Sport, Taichung 404, Taiwan; ^7^Department of Bioresources, Da-Yeh University, Changhwa 515, Taiwan; ^8^The iEGG and Animal Biotechnology Center, and Rong Hsing Research Center for Translational Medicine, National Chung Hsing University, Taichung 402, Taiwan

## Abstract

The mucosa is part of the first line of immune defense against pathogen exposure in humans and prevents viral and bacterial infection of the soft palate, lungs, uvula, and nasal cavity that comprise the ear-nose-throat (ENT) region. Bactericidal/permeability-increasing fold containing family A, member 1 (BPIFA1) is a secretory protein found in human upper aerodigestive tract mucosa. This innate material is secreted in mucosal fluid or found in submucosal tissue in the human soft palate, lung, uvula, and nasal cavity. BPIFA1 is a critical component of the innate immune response that prevents upper airway diseases. This review will provide a brief introduction of the roles of BPIFA1 in the upper airway (with a focus on the nasal cavity, sinus, and middle ear), specifically its history, identification, distribution in various human tissues, function, and diagnostic value in various upper airway infectious diseases.

## 1. Introduction

The gene of the short palate, lung, and nasal epithelium clone 1 (*splunc1*), now referred to as BPIFA1, was first described in the palate and nasal epithelium of murine embryonic and adult lung tissue, which is referred to as* plunk* [1]. The human gene that encodes BPIFA1 contains nine exons that are located on chromosome 20q11.2, and its expression is limited to the upper airway and nasopharyngeal region, including the trachea and nasal epithelium [2, 3]. Di and his colleagues [4] determined that this gene, referred to as* spurt* (secretory protein in upper respiratory tract), was significantly induced by all-trans-retinoic acid in primary cultured human tracheobronchial epithelia.

**Figure 1:**
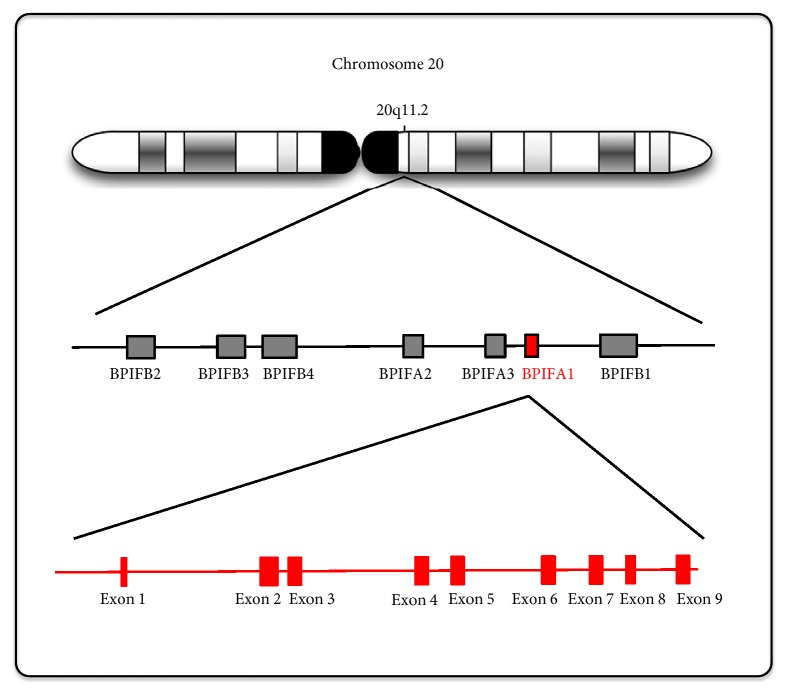
**The genomic location of* BPIFA1* and related* BPI* family members.** *BPIFA1* is located on chromosome 20q11.2 and contains nine exons. There are 7* BPIF* gene families located in loci of* BPIFA1, BPIFA2, BPIFA3, BPIFB1, BPIFB2, BPIFB3,* and* BPIFB4*.

BPIFA1 is a member of a family of seven proteins that are encoded by adjacent genes in an approximately 300 kb region of chromosome 20q11 (Figure 1). The human BPIFA1 cDNA is 1,020 bp in length and contains a leucine-rich protein of 256 amino acids weighing approximately 25 kDa [2]. BPIFA1 is also a member of the parotid secretory protein (PSP)/lipopolysaccharide-binding protein (LBP) superfamily of proteins [5]. Members of the PLUNC family fall into two groups based on their size: “short” proteins, a group comprising BPIFA1 (256 amino acids), SPLUNC2 (249 amino acids) and SPLUNC3 (253 amino acids) and “long” proteins comprising LPLUNC1 (484 amino acids), LPLUNC2 (458 amino acids), LPLUNC3 (463 amino acids), and LPLUNC4 (>469 amino acids) [6]. LPLUNC6/BPIFB6 is important in some virus replication as coxsackievirus B (CVB), poliovirus (PV), and enterovirus 71 (EV71) [7].

SPLUNC proteins contain domains structurally similar to the N-terminal domain of bactericidal/permeability-increasing protein (BPI), whereas LPLUNC proteins contain domains structurally similar to both domains of BPI [6, 8].

Human BPIFA1 is expressed in the salivary gland, and nose of the upper respiratory tract [21]. High expression of BPIFA1 is also found in normal adult nasopharyngeal epithelium [2, 6, 22], trachea, bronchi of the adult lung [1, 23]. Various reports have demonstrated that bactericidal/permeability-increasing fold containing family A, member 1 (BPIFA1) is present in the innate immune material that protects against various diseases and has been widely reported in humans to play a role in lower airway defense against different kinds of bacterial infection. For example, higher levels of BPIFA1 have been correlated with the presence of* Pseudomonas pneumonia* [16, 24, 25],* Klebsiella pneumonia* [26],* Mycoplasma pneumonia* [27], and* Staphylococcus aureus* [28]. BPIFA1 also plays an important role in the innate immunity of the pulmonary airway against influenza A [20, 29] and respiratory syncytial virus infection [14]. Other lower respiratory tract diseases, notably cystic fibrosis, have also demonstrated a correlation between BPIFA1 expression and disease progression [21, 30]. Several reports illustrated a correlation between BPIFA1 expression and middle ear infection [31], chronic rhinosinusitis with nasal polyps (CRSwNP) [32, 33], and sinusitis [34]. Quantitation of BPIFA1 expression could be applied as a diagnostic tool for certain upper airway diseases and may have a value for determining treatment outcomes. Although existing reviews have examined the functions of BPIFA1 and correlations with lower airway diseases, few studies have focused on its functions and correlates in the upper airway.

## 2. BPIFA1 Expression and Regulation

**Figure 2:**
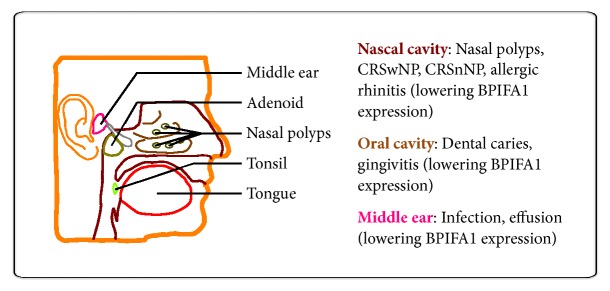
**Highly specific expression of BPIFA1 in the human upper airway respiratory system.** The major expressed tissues of BPIFA1 are located in the tongue, tonsil, nasal polyps, adenoid, and middle ear (as shown in the left panel). Those lowering BPIFA1 expression are affected by infections and correlated diseases of upper airway tracts (as shown in the right panel).

After searching the databases, the BPIFA1 in various upper airway infectious diseases WAS retrieved. Overall, BPIFA1 has been examined through specific expression in upper airway tracts, including the tongue, tonsil, nasal polyps, adenoid, and middle ear (Figure 2).

The materials that affect BPIFA1 expression were summarized to assess future therapeutic applications of this secretory protein in treating upper airway-related diseases (Table 1). Although there are currently no regimens for direct supplementation with BPIFA1, repression of BPIFA1 in patients may still be prevented by controlling or treating the patient's underlying allergic rhinitis or asthma-related disease. This approach may decrease levels of IL-13, a cytokine secreted by many cell types, primarily Th2 helper T cells, to help mediate anti-inflammatory responses to allergens and prevent downregulation of BPIFA1 through the JNK/c-Jun, API, and ERK pathways.

Another critical protein, lactoferrin, has been shown to recover expression of BPIFA1 after LPS-induced infection and is a safe, established product utilized in the treatment of various human diseases [18]. Lactoferrin is a key innate material that defends humans from respiratory syncytial virus, middle ear infections, and sinusitis, while LPS is an endotoxin present in the outer membrane of Gram-negative bacteria that is strongly immunogenic in animals. Supplementation with lactoferrin has been shown to indirectly recover BPIFA1 attenuated by LPS through downregulation of the MEK/ERK pathway, suggesting a possible therapeutic benefit for patients with lower expression of BPIFA1 caused by chronic bacterial infection. Multiple studies have noted that intranasal steroids could restore BPIFA1 to normal levels by reactivating AP-1, the transcription factor responsible for regulating expression of BPIFA1 [35, 36].

Matrix metalloproteinases (MMPs) are responsible for degradation of extracellular matrix (ECM) proteins in normal cell turnover and are upregulated in inflammatory diseases of oral tissues [37], as well as in dental caries and oral cancer [38]. It is hypothesized that elevated MMPs may inhibit the function of BPIFA1. Therefore, inflammation itself could be the reason for downregulation of BPIFA1. Reducing inflammation of the nasal epithelium is another strategy to protect BPIFA1 from inhibition mediated by infection or injury.

How these studies were executed and their materials and methods are shown in Table 2[9, 11–13, 15, 17–19, 39]. Furthermore, Table 3 summarizes the working mechanisms utilized by BPIFA1 to protect humans from related pathogens, viral or bacterial, that infect the upper airway. The primary mode of defense induced by BPIFA1 entails neutralization of LPS by direct binding or action as a surfactant to increase macrophage or neutrophil recruitment to the infected area of the upper airway. These mechanisms are discussed in the following sections.

## 3. Upper Airway Infection and Diseases

Upper airway infections cover a wide disease spectrum in human history from flu virus pandemics to outbreaks of various bacterial infections such as tonsillitis, adenoiditis, sinusitis, pharyngitis, and laryngitis. If not properly controlled or treated, these conditions may be life-threatening. Among infections in the head and neck region, tonsillitis, adenoiditis, and chronic sinusitis are often the most difficult to cure, and they incur major medical costs around the world in both adult and pediatric groups [40]. Various antibiotic-resistant conditions make the diseases hard to treat and susceptible to relapse in current practice due to both the increased population of various penicillin-resistant strains [10] and the intractable condition caused by biofilm accumulation [41]. Therefore, curative surgical procedures such as tonsillectomy and adenoidectomy (T/A) [42], middle ear surgery, and sinus surgery are inevitable and are widely used for chronic or recurrent upper respiratory tract infection (URTI) in child and adult chronic upper airway infection-related diseases. Avoidable surgical treatment options are for patients who have failed to respond to medication, and they are strongly correlated with tissue biofilm formation that subsequently elicits lower BPIFA1 production [16, 17]. Some upper airway infectious diseases such as chronic rhinosinusitis and middle ear infection lead to biofilm formation and lower BPIFA1 expression and require avoidable surgeries. These pathologies are summarized below.

### 3.1. Middle Ear Infection

Current evidence of the direct relationship between middle ear infection and BPIFA1 is lacking. Middle ear effusion, or otitis media, has been shown to exhibit BPIFA1 gene expression [19, 43]. The indirect relationship of otitis media and BPIFA1 may be related to poor eustachian tube function and BPIFA1 expression. Patients with decreased BPIFA1 expression were also noted to be smokers and to present with allergic rhinitis, conditions which were shown to increase the risk of developing otitis media with related middle ear diseases. This decrease in BPIFA1 expression in sinus or upper airway mucosa also leads to repeated infection of the upper airway, including middle ear infection and sinusitis [44].

### 3.2. Sinusitis

Chronic rhinosinusitis comprises a notable portion of upper airway infections and is considered a chronic upper airway infectious disease with diffuse sinus mucosa inflammation. In a case control study, the level of expression of BPIFA1 was depressed in patients with chronic rhinosinusitis, and the protein was considered to have provided a protective function in the sinus mucosa [34]. It was found that sinusitis positive for* Pseudomonas aeruginosa* bacterial culture is associated with decreased BPIFA1 in the sinus mucosa, which may serve as a diagnostic tool to assess expression of BPIFA1 in patients [16]. Furthermore, BPIFA1 expression was found to be significantly reduced in the mucosal epithelia and submucosal glands in patients with multibacterial colonization, particularly those mediated by* Staphylococcus aureus* and* Pseudomonas aeruginosa *[17].

### 3.3. Chronic Rhinosinusitis with Nasal Polyps (CRSwNP)

Patients with chronic rhinosinusitis with nasal polyps (CRSwNP) were found to have lower levels of BPIFA1 expression, which was associated with bacterial colonization [16]. This evidence indicated that decreased levels of BPIFA1 might facilitate bacterial infection in a host, leading to severe disease manifestations. Repeated sinus surgery is correlated with lower BPIFA1 expression and subsequently elevated pseudomonas infection rates. This pattern of repeated surgeries in the sinuses as well as poor surgical outcomes is often observed in CRSwNP [16]. Patients with CRSwNP had higher secretion of interleukin-13 (IL-13), which appears to play a critical role in downregulating BPIFA1 expression. In nasal epithelial cells, IL-13 attenuates BPIFA1 expression by downregulating the lipopolysaccharide- (LPS-) induced activation of phosphorylated JNK and c-Jun [39].

CRSwNP is a disorder characterized by a higher trend of developing Th2-driven inflammation and tissue eosinophilia that may be induced by microbial infection [45]. IL-13, a cytokine predominately secreted by Th2, has been found to contribute to airway allergies and to suppress BPIFA1 expression in nasal epithelial cells [12].

### 3.4. Allergic Rhinitis

The BPIFA1 level was significantly lower in patients with persistent allergic rhinitis (AR) [15, 46]. It was reported that patients with allergic rhinitis had significantly higher chronic rhinosinusitis rates than patients without allergic rhinitis [47]. Patients with lower BPIFA1 expression were found to be more susceptible to sinus infection by certain bacterial infection, which may explain this phenomenon.

## 4. Mechanisms and Therapeutic Use of BPIFA1

### 4.1. Possible Mechanisms of Defense Function

Several studies have shown that BPIFA1 possesses antimicrobial activity and exhibits the same surfactant properties as airway secretions, a trait that may inhibit the formation of bacterial biofilm. It has also been reported that BPIFA1 plays an important role in the regulation of airway surface liquid (ASL) volume [48]. Mechanistically, BPIFA1 contributes to the innate immune response by directly binding to LPS and yielding a bactericidal or bacteriostatic effect against various bacterial infections, an anti-biofilm function, and surfactant properties. Furthermore, BPIFA1 has been shown to be secreted from chemoattracted neutrophils or macrophages to support innate mucosal immunity in the lower airway [25, 49]. It was also reported that BPIFA1 protein binding to bacterial lipopolysaccharide inhibited the growth of* Pseudomonas aeruginosa *[50]*, Klebsiella pneumonia *[26],* Mycoplasma pneumonia *[27], and the Gram-positive bacteria* Staphylococcus areus *[28] by its antimicrobial and surfactant adjusting function.

### 4.2. Mechanism as Surfactant

The surfactant function of BPIFA1 has been well-characterized* in vitro*. BPIFA1 contains hydrophobic residues and is a pH-sensitive regulator that can specifically bind to the epithelial Na^+^ channel (ENaC) [48]. This channel has been shown to be critical to the regulation of ASL and absorption of fluids in many epithelia and is frequently the rate-limiting factor. In addition, BPIFA1 has been hypothesized to act as volume sensors to inhibit ENaC and thus fluid reabsorption by preventing its cleavage and activation by serine proteases. This mechanism inhibits ENaC-dependent Na^+^ absorption, resulting in preservation of airway surface liquid volume. A proteomics screen for molecules that bind trypsin-sensitive ENaC channels identified BPIFA1 as a candidate volume sensor, which was supported by subsequent RNA interference (RNAi) knockdown experiments that demonstrated an inability to regulate ENaC-mediated fluid reabsorption in bronchial epithelial cell cultures [51]. Furthermore, the addition of recombinant BPIFA1 to culture restored ASL volume and ENaC regulation to normal values. Cells coexpressing cystic fibrosis transmembrane conductance regulator (CFTR) and ENaC were incubated with rBPIFA1, which inhibited ENaC activity but did not affect CFTR [52]. This illustrates the specificity of ENaC inhibition by BPIFA1 and its subsequent restoration of ASL, demonstrating a key molecular mechanism underlying the increase in mucosal fluid secretion by elevated BPIFA1 in response to pathogen exposure.

### 4.3. Relation of LPS Interaction

**Figure 3:**
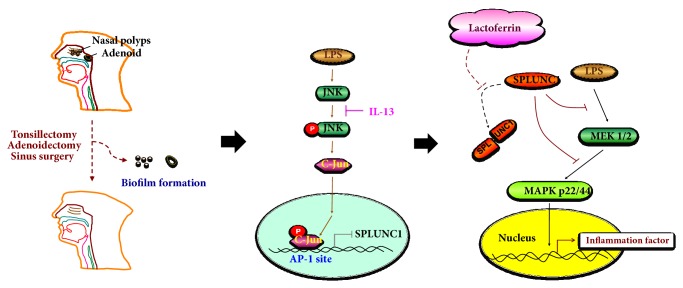
**Proposed mechanism of IL-13 inhibition of LPS-induced BPIFA1 expression in nasal polyps and adenoid tissue.** The IL-13 inhibits* BPIFA1* (*SPLUNC1*) gene expression through a JNK/c-Jun regulation pathway. Lactoferrin also interacts with BPIFA1 in the nasal polyps and adenoid tissues to avoid LPS-induced inflammation via downregulated MEK1/2-MAPK signaling.

Possible biochemical pathways giving rise to anti-pseudomonas and anti-influenza infection may be mapped by surveying elevated chemokines quantitated in BAL and lower airway cell lines or animal lower airway infection models such as higher levels of CXCL1, CXCL2, and CCL20 [50]. CXCL1 recruits neutrophils to the infection site, while CXCL2 recruits monocytes, macrophages, and granulocytes to combat microbial infection by innate immune response. BPIFA1 was found to have an immunomodulatory function through the toll-like receptor 2 (TLR2) pathway [53], which is responsible for recognition of pathogens and recruitment of innate immune cells such as those delineated above. Further, this same review illustrated that downregulation of TLR2 in non-transformed human bronchial epithelial cells through shRNA interference reduced BPIFA1 expression, demonstrating a positive correlation between the activation of this pathway and secretion of BPIFA1 in innate immune material. It was shown that, in defending the lower airway against* Mycoplasma pneumonia* infection, BPIFA1 signaled through the TLR2-NF-*κ*B pathway and also through TLR2-induced (MARK)/activating protein 1 (AP-1) in human lung epithelium cells [54]. In a recent survey using a nasal epithelium (RPMI-2650) model, it was demonstrated that BPIFA1 expression was elevated after LPS-induced inflammation though the MEK/ERK pathway, and BPIFA1 expression was attenuated by IL-13 through the JNK and c-Jun signaling pathway [39] as shown in Figure 3. Lactoferrin was shown to recover the suppressed BPIFA1 through downregulation of the MEK1/2-MAPK signaling pathway by preventing BPIFA1 degradation by LPS. However, whether BPIFA1 exactly could bind with LPS is still questioned till now, since there was also a study which revealed that the LPS is not cognate ligand of BPIFA1 [55]. Besides, we will not just get the concept that BPIFA1 has a similar structure of BPI, which is a major LPS receptor. Actually, there is an allergen called dust mite Der P7 having a similar structure [56]. Therefore, we shall not only just focus on BPI but also check other ligand binding proteins in future studies.

### 4.4. Immune Effects

Neutrophil function was enhanced in the anti-*Mycoplasma pneumonia* immune response induced by BPIFA1 in chronic lung disease [27]. In the lower airway, knockout BPIFA1 mice were shown to have decreased mucociliary clearance and decreased innate immune function [50]. However, this same region in human patients contains human neutrophilic elastase (HNE), which could potentially degrade BPIFA1 in chronic obstructive pulmonary disease (COPD) patients with acute exacerbation of symptoms by nontypeable* Haemophilus influenzae *infection [57]. The mechanism of anti-influenza A function has yet to be elucidated, but it has been shown that increased amounts of BPIFA1 were quantitated after lung and bronchoalveolar lavage (BAL) administration [58]. The protein has been shown to function as an immunomodulatory material in the lower airway, as demonstrated in chronic obstructive pulmonary disease, cystic fibrosis, and idiopathic pulmonary fibrosis. The expression of human BPIFA1 was altered in nasal lavage fluids (NLFs) after exposure to irritants and was quantitated in an increased proportion in NLFs of smokers, suggesting that it is involved in the airway inflammatory response [59, 60].

### 4.5. Microbial Defense

BPIFA1 was also shown to confer a resistance to* Klebsiella pneumonia* infection through its ability to adjust mucosa surface tension in contrast to the BPIFA1 knockout mouse model [26] and to decrease* Mycoplasma pneumoniae* levels that subsequently inhibited epithelial IL-8 production induced by* Mycoplasma pneumoniae*-derived lipoproteins. High levels of BPIFA1 have been shown to be expressed in normal human and mouse large airway epithelial cells. Although* Mycoplasma pneumoniae* infection increases BPIFA1, IL-13 still decreases BPIFA1 expression and* Mycoplasma pneumoniae* clearance, suggesting that BPIFA1 serves as a novel host defense protein against* Mycoplasma pneumonia *[23].

### 4.6. Different Mechanisms and Functions of BPIFA1 between Upper Airway and Lower Airway Infections

The major pathogens present in the lower airway respiratory tract include* Klebsiella pneumonia*,* Pseudomonas aeruginosa*, and* Mycoplasma pneumonia,* while the population of infectious pathogens in the upper airway includes* Staphylococcus aureus*,* Pseudomonas aeruginosa*, and* Klebsiella pneumonia*. Most of the Gram-negative bacilla (GNB) are correlated with LPS toxin-induced infections. BPIFA1 has been shown to neutralize the aforementioned bacterial species in LPS related interaction reported in the lower and upper airways; however, whether BPIFA1 binds to LPS is still questioned [18, 61].

The surfactant function of BPIFA1 is demonstrated mainly in cystic fibrosis in the lower airway and is not widely reported in the mucosa of the upper airway. Furthermore, LPS-induced BPIFA1 expression in the upper airway is mainly involved in JNK/c-Jun [39] and MEK1/2-MARK pathway signaling [18], while the lower airway is mediated by MARK/AP-1 activation through the TLR2 pathway [54]. The lower airway attracted neutrophils and macrophages that could also secrete BPIFA1 and aid in innate immune protection of pulmonary tissue, a phenomenon that has not been reported in the upper airway.

Patients presenting with nasal allergies and Th2-predominant upper airway allergic rhinitis had higher IL-13 expression correlated with low BPIFA1 expression and high sinusitis infection rate than patients lacking a background with allergic rhinitis. This observation also explained a trend in eosinophilic CRS patients who demonstrated higher IL-13 levels and reduced innate immunity to multiple bacterial, and specifically pseudomonas, infections compared to noneosinophilic CRS patients. This pattern was reported in the lower airway in* Mycoplasma pneumonia* clearance related to asthma pathobiology [23].

### 4.7. Materials or Cytokines That Affect BPIFA1 Expression

IL-13 inhibits BPIFA1 expression in upper airway infection. Lactoferrin may recover suppressed BPIFA1 expression by antagonizing LPS and could serve as a future treatment material in nasal spray treatment or sinus irrigation during postoperative care (Table 1).

## 5. Conclusions

The focus of this article was the secretory protein, BPIFA1, which plays a key role in the regulation of airway surface liquid volume and serves in host defense against bacterial infection. BPIFA1 expression was shown to be significantly reduced in the mucosal epithelia and submucosal glands in patients with multibacterial colonization, particularly those mediated by* S. aureus* and* P. aeruginosa*, a trend that may serve as a useful diagnostic marker should methods of BPIFA1 upregulation or supplementation be adapted for therapeutic use. CRSwNP patients requiring repeated sinus surgeries for recurrent or persistent sinusitis also presented much lower BPIFA1 expression than those who did not require repeated sinus surgery. Downregulation of BPIFA1 was shown to produce an immune defect that rendered the host more susceptible to bacterial infection, thus indicating that reduced SPLUNC1 expression might facilitate recurrent* Staphylococcus aureus* and* Pseudomonas aeruginosa* infections in patients with CRSwNP. It was demonstrated that IL-13 plays a critical role in the regulation of BPIFA1 expression in patients by downregulating the LPS-induced activation of phosphorylated JNK and c-Jun, followed by attenuation of BPIFA1 expression in these patients.

Lactoferrin was shown to decrease LPS-induced inflammation-related protein MEK1/2 and p42/44 MAPK activation under BPIFA1 recovery. This finding might suggest that a new therapy could be developed that utilizes lactoferrin treatment to lessen inflammation and increase immune response in persistent infection of host airways.

It was noted that patients who underwent surgical procedures of T/A did not decrease chronic or recurrent upper respiratory tract infection and that repeated bacterial infections in the adenoid were shown to be caused by antibiotic resistance. Furthermore, it was demonstrated that biofilm formation played a major role in these ENT infections. In addition, the colonies of* S. aureus *and* P. aeruginosa* were associated with respiratory diseases, chronic adenoiditis, and rhinosinusitis. Further insight into the mechanism of biofilm resistance raises a useful future direction in infectious diseases and postoperative treatment.

## Figures and Tables

**Table 1:** Materials that affect BPIFA1 expression.

**Effector of BPIFA1 expression**	**Study design materials**	**Correlated diseases**	**Disease findings**	**Mechanism/pathway**	**Biofilm correlation /pathogen**
IL-13	Human nasal lavage fluid	Allergic rhinitis	Lower in allergy	ERK	Not mentioned /not mentioned
	Tissue specimens by sinus surgery	LPS treated RPMI-2650	Lower in polyps tissue	JNK, AP1	Not mentioned/LPS toxin

Lactoferrin	RPMI-2650 cells	LPS treated RPMI-2650	Recovery of BPIFA1	Inhibition of MEK/ERK pathway	Not mentioned /LPS toxin

Intranasal steroid	RPMI-2650 cells	LPS treated RPMI-2650	Recovery of BPIFA1	Reactivation of AP-1	Not mentioned /LPS toxin

MMP9 (matrix metallo-proteinase 9)	Targets cells/macrophages/oral epithelia/airway epithelia	PLUNCS inhibited by MMP9	PLUNCS inhibited	PLUNC protein proteolytically cleaved by MMP9	Not mentioned /microorganism colonization

**Table 2:** Summary of available BPIFA1 studies, methods of analysis utilized, and related upper airway diseases under study.

**Author (year)/[Reference]**	**Study design**	**Material**	**Methods**	**Correlated upper airway diseases**
Casado et al. (2005)/[9]	Human (in vivo)	Nasal lavage fluids	Proteomics	Upper airway microbial infections and unclean inhaled air

Tsou et al. (2015)/[10]	Human (in vitro)	Septum squamous carcinoma	Western blot, RT-PCR, IHC for expression levels of BPIFA1 and IL-13	Human chronic rhinosinusitis with nasal polyps

Bingle and Gorr (2004)/[11]	Human (in vitro)	Human oral, nasal and respiratory epithelia	Cell line, genomes sequencing, cDNA, cDNAarray etc.	Oral, respiratory, GNB infection

Yeh et al. (2010)/[12]	Human (in vitro)	Nasal polyp epithelial cells in air-liquid interface culture	RT-PCR, Western blot	CRSWNP (chronic rhinosinusitis with nasal polyps)

Fornander et al. (2013)/[13]	Human (in vivo)	Nasal lavage fluids	Western blot, 2D gel electrophoresis	Upper airway symptoms

Fornander et al. (2011)/[14]	Human (in vivo)	Naspharyngeal aspiration	2D gel electrophoresis and mass spectrometry	Respiratory syncytial virus infection

Ghafouri et al. (2006)/[15]	Human (in vivo)	Nasal lavage fluids	2D gel electrophoresis, and matrix assisted laser desorption/ionization time-offlight mass spectrometry	Seasonal allergic rhinitis

Tsou et al. (2013)/[16]	Human (in vivo)	Sinus polyps	Bacterial culture, RT-PCR, IHC	CRSWNP (chronic rhinosinusitis with nasal polyps)

Tsou et al. (2014)/[17]	Human (in vivo)	Sinus polyps	Bacterial culture, RT-PCR, IHC	Sinonasal infections, CRSWNP

Tsou et al. (2017)/[18]	Human (in vitro)	Nasal RPMI-2650 cells	Western blot, indirect immunofluorescence, confocal laser scanning microscopy, and quantitative fluorescence analysis	CRSWNP (chronic rhinosinusitis with nasal polyps)

Rye et al. (2012)/[19]	Human (in vivo)	Genomic DNA was extracted from blood	Single nucleotide polymorphisms	Otitis media

Teran et al. (2012)/[20]	Human (in vivo)	Nasal aspirates	2D gel electrophoresis and mass spectrometry	Seasonal influenza A virus

**Table 3:** Summary of mechanisms, correlated pathogens, and findings from studies of BPIFA1 and related upper airway diseases.

**Author (year)/[Reference]**	**Mechanism**	**Pathogen**	**Findings**
Casado et al. (2005)/[9]	Innate immune response in the nose against microbial infections and unclean inhaled air	Not mentioned	LC-ESI-MS/MS was involved in acquired and innate immune response in the nose against microbial infections and unclean inhaled air, e.g., rhinosinusitis

Tsou et al. (2015)/[10]	IL-13 perturbation of GNB-related bacterial infection and BPIFA1 expression in CRSwNP through JNK/c-Jun pathway	Lipopolysaccharide (LPS) related GNB such as *Haemophilus influenza*, *Pseudomonas aeruginosa*, *Klebsiella pneumonia*	IL-13 attenuated LPS (GNB) bacteria-induced BPIFA1 expression causing compromise of certain GNB bacterial infections

Bingle and Gorr (2004)/[11]	LPS neutralization	Not mentioned	PLUNC proteins mediate host defense functions in the oral, nasal and respiratory epithelia

Yeh et al. (2010)/[12]	Inhibition of BPIFA1 production	Not mentioned	IL-13 is harmful to the host innate immune response through the inhibition of BPIFA1 production

Fornander et al. (2013)/[13]	BPIFA1 is a target of human neutrophil elastase (HNE) activity	Metal working fluids (biocides, surfactants, anti-oxidants and corrosion inhibitors)	IL-1b was significantly higher in subjects with airway symptoms

Fornander et al. (2011)/[14]	Innate immune response	Respiratory syncytial virus	A decrease in BPIFA1 in the upper airways may increase the risk for severe pneumonia

Ghafouri et al. (2006)/[15]	NLF levels of the cysteine proteinase inhibitors, cystatin S and VEGP were decreased and failed to inhibit proteinase action in SAR	Pollen	BPIFA1 is significantly decreased in the NLF during nasal inflammation by allergic rhinitis

Tsou et al. (2013)/[16]	Anti-bacteria or surfactant	*Pseudomonas aeruginosa*	Sinusitis with bacterial culture positive for *P. aeruginosa *isassociated with lower expression of BPIFA1 and unfavorable sinus surgery outcomes

Tsou et al. (2014)/[17]	Anti-bacteria or surfactant	GPC: *Staphylococcus areus* GNB: *Pseudomonas aeruginosa*, *Haemophilus influenza*, *Klebsiella pneumonia*	BPIFA1 is a novel predictive outcome biomarker for patients with CRSwNP and bacterial colonization Lower BPIFA1 related to multiple bacterial infections

Tsou et al. (2017)/[18]	Lactoferrin increases BPIFA1 expression to regulate inflammation in RPMI-2650 cells through JNK/cJun pathway	Gram-negative bacteria	Lactoferrin could be a possible treatment strategy for LPS-induced chronic rhinosinusitis

Rye et al. (2012)/[19]	Genome-wide associated study of childhood otitis media susceptibility found that decreased BPIFA1 was correlated with higher otitis media in children	Not mentioned	GWAS was performed to identify the genetic determinants of OM in childhood

Teran et al. (2012)/[20]	Lipocalin-1 can enhance the bactericidal activity of lysozyme and exhibit inherent antimicrobial function	Seasonal influenza A virus	To provide the pathogenesis of respiratory infections caused by seasonal influenza A virus in nasal fluid

## Abbreviations

AR: Allergic rhinitis
ASL: Airway surface liquid
BPIFA1: Bactericidal/permeability-increasing fold containing family A, member 1;
CFTR: Cystic fibrosis transmembrane conductance regulator
COPD: Chronic obstructive pulmonary disease
CRSwNP: Chronic rhinosinusitis with nasal polyps
ECM: Extracellular matrix
ENaC: Epithelial Na+ channel
ENT: Ear-nose-throat
GNB: Gram-negative bacilla
HNE: Human neutrophilic elastase
IHC: Immunohistochemistry
LBP: Lipopolysaccharide-binding protein
MMPs: Matrix metalloproteinases
NLFs: Nasal lavage fluids
PSP: Parotid secretory protein
SPLUNC1: Short palate, lung, and nasal epithelium clone 1
TLR2: Toll-like receptor 2
URTI: Upper respiratory tract infection.
